# Bumblebee foraging rhythms under the midnight sun measured with radiofrequency identification

**DOI:** 10.1186/1741-7007-8-93

**Published:** 2010-06-29

**Authors:** Ralph J Stelzer, Lars Chittka

**Affiliations:** 1 School of Biological and Chemical Sciences, Queen Mary University of London, Mile End Road, London, E1 4NS, UK

## Abstract

**Background:**

In the permanent daylight conditions north of the Arctic circle, there is a unique opportunity for bumblebee foragers to maximise intake, and therefore colony growth, by remaining active during the entire available 24-h period. We tested the foraging rhythms of bumblebee (*Bombus terrestris *and *B. pascuorum*) colonies in northern Finland during the summer, when the sun stays above the horizon for weeks. We used fully automatic radio-frequency identification to monitor the foraging activity of more than 1,000 workers and analysed their circadian foraging rhythms.

**Results:**

Foragers did not use the available 24-h foraging period but exhibited robust diurnal rhythms instead. A mean of 95.2% of the tested *B. terrestris *workers showed robust diurnal rhythms with a mean period of 23.8 h. Foraging activity took place mainly between 08:00 and 23:00, with only low or almost no activity during the rest of the day. Activity levels increased steadily during the morning, reached a maximum around midday and decreased again during late afternoon and early evening. Foraging patterns of native *B. pascuorum *followed the same temporal organisation, with the foraging activity being restricted to the period between 06:00 and 22:00.

**Conclusions:**

The results of the present study indicate that the circadian clock of the foragers must have been entrained by some external cue, the most prominent being daily cycles in light intensity and temperature. Daily fluctuations in the spectral composition of light, especially in the UV range, could also be responsible for synchronising the circadian clock of the foragers under continuous daylight conditions.

## Background

Bumblebee colonies are typically founded in spring by individual fertilised queens and are subsequently engaged in a race against time to maximise colony growth and ultimately the production of more queens and males while favourable conditions last [1,2]. In temperate regions, therefore, the first bumblebee workers typically leave the nest to forage as soon as it is bright enough, often even at dawn before the actual sunrise and the last foraging trips of the day are concluded around sunset [3]. The foraging activity of the workers is synchronized to the available photoperiod by endogenous circadian clocks [4,5].

Given that foragers use the entire available photoperiod in temperate regions, one would expect that they would also fully utilise longer or even continuous photoperiods prevailing north of the Arctic Circle during summer to gather as much food as possible and thus maximise colony growth. Other animal species in that area, such as reindeer, become arrhythmic during the summer, when the sun is continuously above the horizon [6,7]. Previous studies have found an impressive sociality-dependent plasticity in the circadian rhythms of social insects, with the ability to alter the expression of clock genes to fit the demands of the colony [8-10]. Hence we might expect that arctic bumblebees would adapt to the foraging conditions that prevail under the midnight sun.

To answer the question whether bumblebee foragers utilise the available photoperiod under continuous daylight conditions, we conducted field experiments with free-flying bumblebees during midsummer in northern Scandinavia. The sun did not set for weeks during our experiments, giving the foragers the opportunity to forage around the clock. We used fully automatic radio-frequency identification (RFID) to monitor the foraging activity of hundreds of individual *B. terrestris *workers around the clock for several weeks and analysed their circadian foraging rhythms. Since *B. terrestris *is not native that far north, we also recorded the traffic at the nest entrance of a native *B. pascuorum *nest.

## Results

A total of 1,049 workers were radio-tagged in our experiments. The RFID readers never recorded 164 of these workers leaving the nest, indicating that these workers spent their time entirely with tasks inside the nest. The data generated by the remaining 885 workers were used for the analysis of the circadian foraging rhythms on the colony level. On the individual level, the data of another 504 workers were discarded from the circadian analysis because they did not meet the requirement of at least six consecutive days of activity, leaving a total of 381 workers for individual analysis.

**Figure 1:**
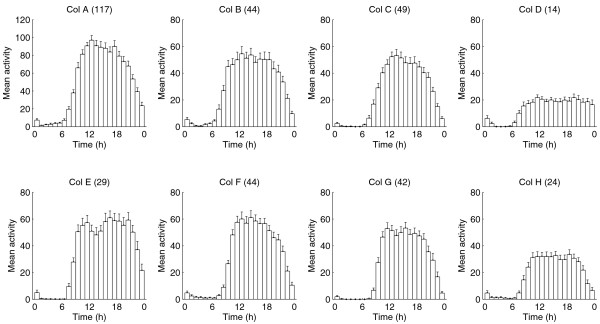
**Daily average plots of the foraging activity under continuous daylight conditions in the field**. The top row shows daily average plots of the *Bombus terrestris *colonies observed at Kilpisjärvi Biological Station, Finland, in the summer of 2007 and the bottom row shows the data for the colonies observed in the summer of 2008 at the same location. Numbers in brackets indicate the number of tagged workers meeting the requirements to be included in the circadian analysis per colony. Each bar represents an hour of the day and the height of the bars indicates the level of activity.

**Figure 2:**
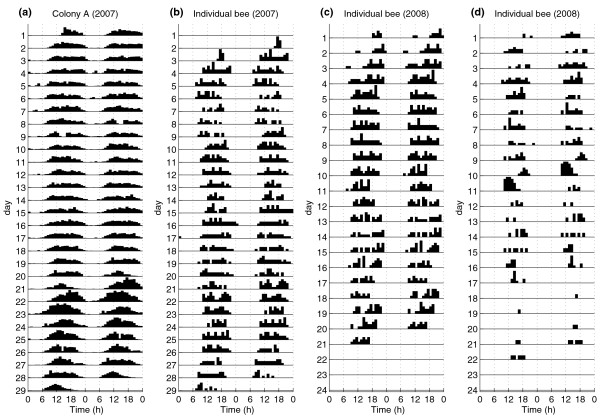
**Exemplary double plotted actograms of a colony and individual foragers**. Double-plotted actogram of **(a) **a *Bombus terrestris *colony (colony A) and **(b) **an individual worker of that colony. **(c) **and **(d) **actograms of two individual foragers of colony F to highlight the differences in foraging activity of individual workers. Each line represents two consecutive days of the experiment, each bar indicates 1 h of the day and the height of the bars indicates the level of foraging activity in that hour.

**Figure 3:**
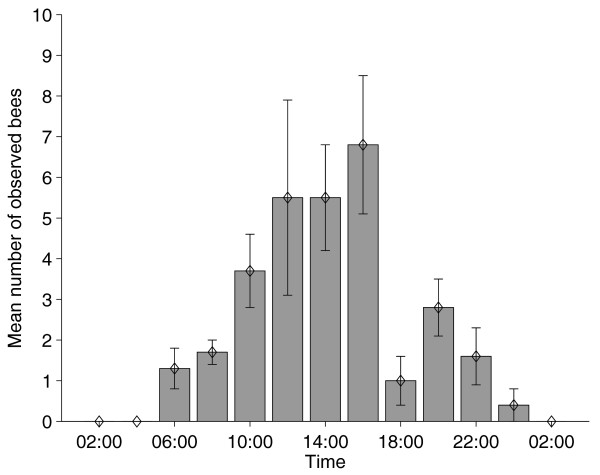
**Mean traffic at the nest entrance of a natural *Bombus pascuorum *nest**. The nest was located near the field station and traffic was monitored for 30 min in intervals of 2 h. Every leaving or returning bee was counted.

Although bumblebees experienced 24 h of bright daylight in the field, foraging activity was restricted mainly to the times from 08:00 to 23:00, with almost no activity between 01:00 and 06:00 (Figure 1). Activity levels started to increase between 06:00 and 07:00, rising steadily during the morning and reaching the maximum during midday before they decreased again (Figure 1). Thus, the colonies exhibited a robust diurnal rhythm in their foraging patterns (Figure 2). The same was true on the individual level. A mean of 95.2% ± 1.2% (mean ± 1 SEM) of individual workers showed a robust diurnal rhythm in their foraging activity, with a mean period of 23.8 h ± 0.04 h (Figure 2). The foraging pattern of the native *B. pascuorum *colony was similar to the ones observed in the introduced *B. terrestris *colonies, with the foraging activity being restricted to the period between 06:00 and 22:00 and no recorded activity during the 'night' (Figure 3).

In 2007, light intensity was measured between 1 July 2007 and 8 July 2007. In that period, the hourly mean values varied between 2,670 lux ± 520 lux at 02:00 and 162,110 lux ± 6,490 lux at 13:00. Individual measurements reached a maximum of 275,560 lux on a sunny day and dropped below 1,000 lux only during one cloudy night with a minimum of 820 lux. Mean hourly ambient temperature in the same time period varied from 9.9°C ± 0.7°C at 02:00 to 18.4°C ± 1.3°C at 12:00, with individual measurements ranging from 6.6°C to 23.9°C and a mean daily fluctuation of 9.8°C ± 0.9°C.

**Figure 4:**
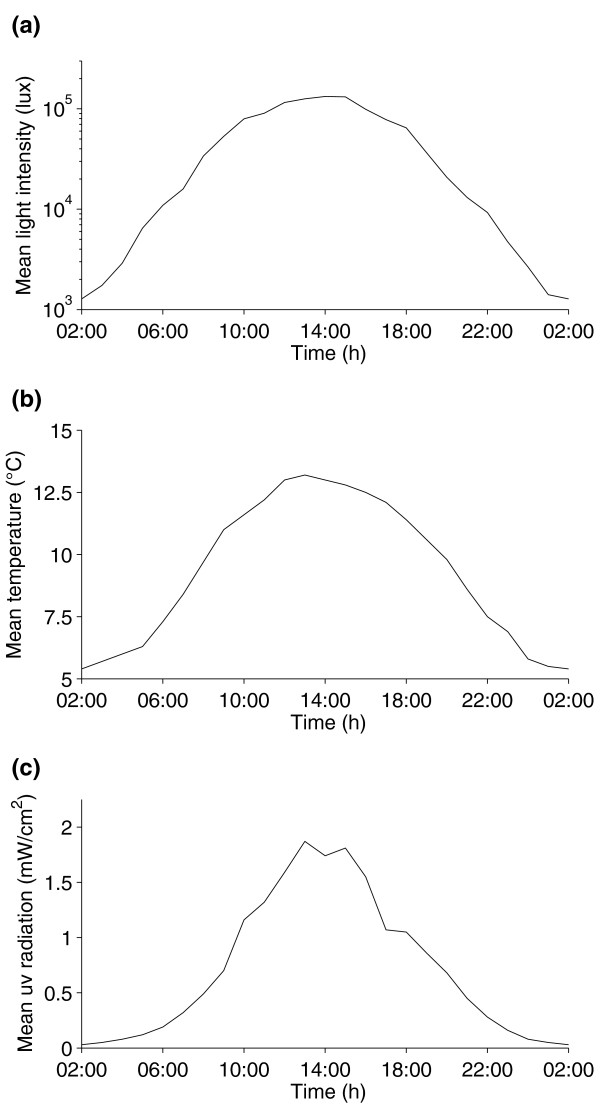
**Mean hourly values of environmental cues that could act as possible Zeitgebers on the circadian clocks of bumblebees under continuous daylight conditions**. **(a) **light intensity, **(b) **ambient temperature and **(c) **UV radiation (320 nm to 410 nm) recorded between 5 July 2008 and 13 July 2008 at the field station. Two days (7 July 2008 and 8 July 2008) have been excluded from the UB measurements because the values were not recorded for the whole 24 h of these days. Temperature data were obtained from the Finnish Meteorological Institute and UV data were kindly provided by Dr. Iris Zellmer.

In 2008, light intensity was measured from 5 July 2008 to 13 July 2008. The hourly mean light intensity varied between 1,280 lux ± 110 lux at 02:00 and 132,650 lux ± 17,750 lux at 14:00 (Figure 4a). Individual measurements stretched from 740 lux to 192,980 lux. Light intensity dropped below 1,000 lux during two nights only. Mean hourly ambient temperature ranged from 5.4°C ± 0.5°C at 02:00 to 13.2°C ± 0.8°C at 13:00 (Figure. 4b). Individual measurements varied from 3.6°C to 18.2°C, with a mean daily fluctuation of 8.3°C ± 0.5°C.

## Discussion

Our results show that bumblebee foragers do not utilise the 24-h foraging period under constant daylight conditions in northern Scandinavia but exhibit robust diurnal rhythms in their foraging patterns instead. In previous carefully controlled laboratory experiments [4], foragers showed free-running circadian rhythms both in constant light conditions (LL) and constant darkness (DD), with mean free-running periods (τ) being significantly shorter in LL than in DD (LL: τ = 22.4 h at about 2,300 lux; DD: τ = 24.0 h). Free-running circadian rhythms in laboratory LL conditions have previously also been found in the related honeybee (*Apis mellifera*) [11-13]. The rhythmic behaviour of bees under LL conditions is in contrast to findings in the fruit fly *Drosophila melanogaster*, a model organism for chronobiological studies [14-16], which become completely arrhythmic in LL [17]. In *Drosophila*, the blue-light receptor cryptochrome (dCRY) is largely responsible for the photic entrainment of the circadian clock [18-21]. The decoding of the honeybee genome revealed that honeybees lack a *Drosophila*-type CRY but encode a vertebrate-like protein (CRY2) instead [22]. CRY2 has also been found in other insect species, such as butterflies and mosquitoes [23], as well as in the North American bumblebee *Bombus impatiens *[24]. In contrast to dCRY, CRY2 is not photosensitive [24] and thus might not function as a circadian photoreceptor, which poses the yet unanswered question how light enters the circadian system of bees.

The fact that the bees did not show free-running rhythms in the prevailing continuous daylight conditions indicates that some external cue, or Zeitgeber, must have entrained the molecular clock of the bees in the field, the most prominent cue being natural cycles in light intensity and associated temperature fluctuations. Although the sun always stayed above the horizon during our experiments, levels of light intensity varied drastically during the course of the day. Laboratory studies have shown that, on average, a minimum of 3.2 lux is needed for *Bombus terrestris *workers to be able to fly [25] and they have been observed leaving the nest at light intensities below 50 lux on summer mornings in Germany (RJS, unpublished data). During most '"nights"' of our experiments, light levels stayed well over 1,000 lux during the darkest part (Figure 4a) and fell below 800 lux only during the last week of the experiment. Thus it was never too dark for the bumblebees to forage, however the gradually decreasing light levels in the evening could have triggered the daily resetting of the circadian clock of the foragers and studies on *Drosophila *have shown that the absolute levels of light are less important than the relative light/dark light intensities for rhythm entrainment [26,27].

In line with the changes in light intensity there were daily cycles in ambient temperature (Figure 4b). Although circadian rhythms are normally temperature compensated [28], circadian clocks can be synchronised by temperature cycles. In *D. melanogaster *and the honeybee, daily temperature cycles of just 3°C and 6°C, respectively, are enough to entrain circadian rhythms in their locomotor activity [29-32]. Thus, daily temperature cycles during our experiments were in a range where temperature entrainment might be possible, although experiments on arrhythmic mutant *Drosophila *flies have shown that a functional circadian clock is indeed needed for the anticipation of daily temperature changes but is not essentially required for the generation of diurnal activity rhythms under such conditions [33]. The minimum temperatures recorded in our field experiments were as low as 3.6°C during exceptionally cold nights. However, *Bombus terrestris *workers have been observed foraging at ambient temperatures as low as 3°C during late winter in London, UK, and the foraging rates they achieved under these conditions were similar to those found during summer [34]. Therefore, the temperature during our experiments did not drop below a threshold that would have made it impossible for the bumblebees to forage and the foragers should have been able to gain a net energy profit in these temperatures.

Another possible Zeitgeber are daily cycles in light quality, i.e. in the spectral composition of light, especially in the UV range. Daily changes in ultraviolet radiation can be used to entrain and influence the circadian rhythms in other species [35]. Figure 4c shows the hourly means of UV-A radiation (320 nm to 410 nm) recorded at the field station during seven days of our experiments in 2008. Like other bees, bumblebees are UV-blue-green trichromats and can thus see UV light [36,37]. A recent study in bumblebees has found a new photoreceptor organ that expresses UV-sensitive opsins in a brain area that has been associated with circadian clock activity in other insects [38], so it might be possible that the circadian clock of bumblebees can be entrained by daily cycles in UV radiation levels [39].

Finally, daily variance in the availability of floral resources could have been another Zeitgeber. However, in that case we would expect that the foragers would carry on searching for food during the first few nights after being set up in the field before they gradually adjust their foraging patterns to the diurnal pattern of resource availability, which was not the case.

The native *B. pascuorum *colony observed at the field station showed the same behaviour as the introduced *B. terrestris *colonies, indicating that our results were not an artefact of using a temperate species in permanent daylight conditions. Field observations at flower patches during summer nights in northern Scandinavia also found a drop in foraging activity around midnight [40]. Thus it seems that even native bumblebees are not adapted to fully use the whole available photoperiod, which is surprising given that the vegetation period at these latitudes is quite short and colonies have relatively little time to grow. Reindeer (*Rangifer tarandus*) have adapted to these extraordinary conditions by loosing their molecular circadian clock [41]. But staying inside the nest during the nights, although it is bright enough to forage, could be an adaptive behaviour, especially for native bumblebees since colonies in the far north are generally quite small [42] and the foragers might therefore be needed inside the nest during the cooler nights to warm the brood. The cold nights might especially affect species like *B. pascuorum*, which build their nests on or just below the soil surface [43]. Another possible adaptive reason for extended resting periods might be positive effects on the memory of the foragers. Previous studies have found a mammalian-like sleep behaviour in honeybees [44,45]. Honeybees compensate for sleep deficit by intensifying the sleep process during the following night [46], which makes it unlikely that they show this sleep behaviour just to save energy during the night and it also has been shown that sleep deprivation affects the consolidation of memory in bees [47].

## Conclusions

Our results show that bumblebee foragers do not use the whole 24 h foraging period under the continuous daylight conditions that prevail north of the Arctic Circle during summer. Foragers show robust diurnal rhythms under these conditions, which means that their circadian clocks must be entrained by some external cue. The fact that even foragers of bumblebee species native to the Arctic are inactive during the '"night"' indicates that this might be an adaptive behaviour. More studies are needed to identify the adaptive reasons for the observed foraging patterns and the Zeitgeber for synchronising the circadian clock of the foragers under continuous daylight. There might also be a synergism between several external cues as has been suggested previously for light intensity and temperature in honeybees [30].

## Methods

### Study species and location

Field experiments were conducted during the summers of 2007 (20 June 2007 to 18 July 2007), 2008 (29 June 2008 to 23 July 2008) and 2009 (12 July 2009 to 21 July 2009) at Kilpisjärvi Biological Station in northwestern Finland (69° 03'N, 20° 50'E; 473 m a.s.l.). The station is located 270 km north of the Arctic Circle, so there was continuous sunshine from 22 May to 22 July in that area. Mountain birch forests and heaths, as well as marshland, dominate the flora. The main flower species visited by bumblebees include *Astralagus alpinus*, *Bartsia alpina*, *Salix *spp., *Saxifraga oppositifolia*, *Silene dioica *and *Vaccinium *spp. [48,49]. In both 2007 and 2008 four *Bombus terrestris (*subspecies *terrestris*) colonies were tested simultaneously using radio-frequency identification (see below). The colonies were obtained from commercial breeders (2007: Koppert Biological Systems, Berkel en Rodenrijs, The Netherlands; 2008: Syngenta Bioline Bees, Weert, The Netherlands) shortly before they were set up in the field. The colonies were housed in bipartite plywood nest boxes (28 cm × 16 cm × 11 cm) that were placed in sheds at and near the field station and covered with fleece blankets. The boxes were connected to the outside by short transparent Plexiglas tunnels with a system of shutters to enable movement of bees into and out of the nest to be controlled by the observer. During the experiments, all workers were allowed to leave the nest at will. Before the colonies were placed in the field, they were fed *ad libitum *with sugar solution and pollen that came with the colonies. Since *B. terrestris *is native in southern Scandinavia but not that far north, males and new queens (gynes) were removed from the colonies to prevent establishment of this species at the study location due to our experiments. Light intensities were recorded using a data logger whose sensor was facing straight up and not shielded from direct sun to be able to get unflawed measurements of the minimum light levels during the 'night' (HOBO Temperature/Light Pendant Data Logger, UA-002-64; Onset Computer Corporation, Pocasset, MA, USA). Ambient temperature data from the study location were obtained from the Finish Meteorological Institute. UV-A radiation levels (320 nm to 410 nm) at the field station in 2008 were kindly provided by Dr. Iris Zellmer (Institute of Plant Physiology, Martin-Luther-University Halle, Halle/Saale, Germany). In 2009 traffic at the nest entrance of a native *Bombus pascuorum *colony was monitored for 30 min in intervals of 2 h and every leaving or returning worker was counted.

### Radio-frequency identification

To be able to monitor the complete foraging activity of individual bees, RFID was used [4,34,50,51]. Small RFID tags (mic3^®^-TAG 64-bit RO, iID2000, 13.56 MHz system, 1.0 mm × 1.6 mm × 0.5 mm; Microsensys GmbH, Erfurt, Germany) were glued to the dorsal surface of the thorax of the bees. An RFID reader (iID2000, 2k6 HEAD; Microsensys GmbH, Erfurt, Germany) was integrated into the tunnel close to the nest entrance. The RFID reader automatically recorded the date and time when a tagged worker passed it, as well as the identity of the passing bee. The data were downloaded from the RFID readers every day.

### Data analysis

The level of activity was analysed in 60-minute bins. The resolution on the individual level was chosen as 1 min, i.e., for each hour the number of minutes in which a given tagged bee passed the reader at least once was counted and used as the level of activity within that hour. The raw data downloaded from the RFID readers were processed accordingly, using macros (Virtual Basic for Applications) developed by the authors using Microsoft Excel. The processed data were then used for the circadian analysis at both the colony and the individual level in MATLAB (version R2007a; The Mathworks, Natick, MA, USA) [4,52]. For the analysis on the colony level, all available processed data were used. On the individual level, only bees that were active during at least six consecutive days were analysed further.

Actograms and daily average histograms were plotted using MATLAB. The statistical significance of circadian rhythms was assessed using rhythmicity statistics (RS), which provide a numerical accounting of significance [53]. The RS value for each individual bee was obtained through autocorrelation analysis performed using MATLAB [52]. Bees with an RS value of 1 or above were considered rhythmic (see [53] for how this cutoff was determined). The free-running periods were calculated using Maximum Entropy Spectral Analysis [52,53].

## Authors' contributions

LC and RJS conceived of the study and designed the experiments. RJS carried out the experiments and data analysis. RJS and LC drafted the manuscript. All authors read and approved the final manuscript.
